# Natural History Characterization and Biological Observations of *Neobrachelia edessae* (Diptera: Tachinidae), an Antagonist Fly of the Stink Bug Pest *Edessa meditabunda* (Hemiptera: Pentatomidae)

**DOI:** 10.1007/s13744-026-01396-5

**Published:** 2026-04-30

**Authors:** María Candela Barakat, Rodrigo de Vilhena Perez Dios, Sofía Pilar Díaz, María Fernanda Cingolani

**Affiliations:** 1 Centro de Estudios Parasitológicos y de Vectores (CEPAVE) (CONICET-UNLP-Asoc. CICPBA), La Plata, Argentina; 2 Lab de Diptera, Museu de Zoologia, Univ de São Paulo, São Paulo, Brazil

**Keywords:** Biocontrol agent, Natural history, Pentatomidae, Self-superparasitism, State-dependent parasitism, Tachinidae

## Abstract

Natural enemies are a good alternative for pest control. To enhance their role as biocontrol agents, understanding their biology and ecology is necessary. Pentatomid hemipterans are pests of important crops, and tachinids are natural enemies of their adult stage. *Neobrachelia edessae* (Diptera: Tachinidae) is a Neotropical parasitoid fly of the stink bug *Edessa meditabunda* (Hemiptera: Pentatomidae), with practically unknown biology. We evaluated the life history of this parasitoid when developing on adults of *E. meditabunda*, and described the morphology of larval instars. Differences in developmental times and longevity were evaluated considering the number of parasitoid larvae per host, sex of the parasitized host and sex of the fly offspring. Egg to pupa development time was affected by the number of larvae developing within the host. On average, 27 days were needed to complete larval development if there was a single larva per host, whereas 30 days on average were needed when more than one larva developed within a host. Pupa to adult development time was on average 19 days, and average adult longevity was 11 days. Developmental time and longevity of parasitoid offspring were not affected by either host sex or parasitoid sex. Given that biological information for this species is scarce, as is the case for most tachinid species, these results are relevant for the design of biological control programs in the future.

## Introduction

Stink bugs (Hemiptera: Pentatomidae) are important pests of several crops worldwide (Conti et al. 2021), and agriculture is a key economic activity in Latin America (Mohammadi et al. 2020; de Salvo et al. 2025), with soybean being one of the most affected commodities. Massification of this crop has been accompanied by increases in stink bug populations and pesticide use (de Aquino et al. 2020), leading to resistance and failures in stink bug control (de Aquino et al. 2020; Pajač Beus et al. 2024). Therefore, biocontrol solutions are increasingly in demand, aligned with integrated pest management (IPM) strategies (Stenberg et al. 2021). Within this framework, biocontrol consists in the use of antagonistic organisms, such as parasitoids, to reduce pest populations in an environmentally safe and economically profitable manner (van Lenteren et al. 2020).

Tachinid flies (Diptera: Tachinidae) are the largest group of non-hymenopteran parasitoids. They are extremely diverse, with rapid diversification, but are often poorly studied (Stireman et al. 2006, 2021; Dindo et al. 2019). Flies of the subfamily Phasiinae attack hemipterans, many of which are considered agricultural pests (Blaschke et al. 2018), and are important controllers of stink bugs (Duncan 2017; Lucini et al. 2020). However, relationships between Phasiinae flies and their hosts remain poorly known (Liljesthröm and Ávalos 2015; Fernández et al. 2024), and more studies are necessary on their taxonomy, ecology and biological characteristics (Dindo et al. 2019; Stireman et al. 2019). The establishment of laboratory rearing for tachinids is challenging, largely due to this lack of biological information (Dupuis 1963; Dindo et al. 2019). This knowledge gap is particularly evident in the genus *Neobrachelia* which comprises four valid species (O’Hara et al. 2020) and differs morphologically from most phasiines by having many setae on their bodies and distinctive terminalia.

*Neobrachelia edessae* is a parasitoid of *Edessa meditabunda* and *Edessa rufomarginata* (Hemiptera: Pentatomidae) (Guimarães 1977) and has been recorded in Uruguay (Townsend 1942; Parker 1953) and Argentina (Barakat et al. 2023; Fernández et al. 2024). However, its biology remains unknown. Only host records and illustrations of the cephalopharyngeal skeleton of the first larval instar and the puparium posterior spiracles have been reported (Parker 1953). Although tachinids lack the perforating ovipositors of hymenopteran parasitoids, they employ different oviposition strategies: indirect oviposition when eggs are laid in the environment near to the potential hosts, or direct oviposition when eggs are laid on or inside the host (Dindo and Grenier 2023). In this sense, some tachinids have developed a piercing structure (Dindo and Grenier 2023) that allows them to lay eggs internally within the host body. This oviposition behaviour may contribute to the underestimation of parasitism, since no external signs of parasitism are observed. However, records of *N. edessae* parasitising adults of *E. meditabunda* in the region are common (Barakat et al. 2023; Fernández et al. 2024). The host is also distributed in the Neotropical region in Argentina, Brazil, Bolivia, Colombia, Paraguay, French Guiana, and Uruguay in South America, and in Antigua and Barbuda, Cuba, the Dominican Republic, Trinidad and Tobago, and Saint Vincent and the Grenadines in the Caribbean region (Panizzi 2015, Dellapé et al. 2025). It is considered a major pest of soybean crops, but its host range is much broader than for other species in the complex. It attacks various commercially important crops such as cotton, eggplant, tobacco, sunflower, papaya, tomato, potato, alfalfa and grapevine (Silva et al. 2021; Panizzi et al. 2022).

Several aspects are relevant when establishing a parasitoid colony, some related to abiotic factors, such as temperature, humidity and photoperiod, while others are related to biotic factors, including diet, mating behaviour, oviposition strategies and host range (Dindo and Grenier 2023). In particular, host quality is an important factor in laboratory rearing of parasitoids, as it may affect the decision to parasitize or not (van Lenteren 2003). Moreover, as the diet of the host influences its quality (Reitz and Trumble 1997), it is important to determine whether laboratory-reared hosts, which develop under different conditions than field-reared hosts, are suitable for maintaining a colony of natural enemies.

Parasitoid behaviours are state-dependent. For example, oviposition decisions depend on the female egg load, feeding decisions depend on nutritional state, host discrimination can be a function of information acquisition and patch leaving decisions can be influenced by parasitoid age (Roitberg and Bernhard 2008). Parasitoids can assess internal and environmental variables and adjust their clutch size accordingly to maximize their reproductive fitness (Godfray et al. 1991). Self-superparasitism occurs when a host is parasitized multiple times by the same female (van Alphen and Visser 1990), a frequent behaviour in tachinids (Beckers 2022). Understanding the adaptive nature of parasitoid behaviours can help to exploit these traits to maximize the fitness of these biological control agents in the agroecosystems.

In this context, intrinsic competition (i.e. competition among immature parasitoids developing in the same host) can affect offspring performance (Harvey et al. 2013; Mohamad and Goubault 2023). If a larva consumes less food due to intrinsic competition or poor host quality, its energy reserves will be lower, and development may be affected (van Lenteren 2003). Therefore, in solitary parasitoids, auto-superparasitism, defined as multiple attacks of a host by the same parasitoid female, can negatively affect the performance of her offspring (Mohamad and Goubault 2023). In addition, host characteristics, such as sex, can influence the resource available to parasitoid larvae, because body size and internal structure may differ between sexes (Araújo et al. 2020). Moreover, the sex of the parasitoid offspring may have different energy requirements to successfully complete their life cycle (Kalyebi and Nakamura 2006; Caron et al. 2010).

The main objective of this work is to improve knowledge of the biology of this parasitoid fly. In particular, developmental times, longevity and morphological characteristics of different stages of the life cycle of *N. edessae* when developing on adults of *E. meditabunda* as hosts are evaluated and described.

## Materials and Methods

Insect colonies were established from field-collected individuals from La Plata city (Argentina, −35.056307, −57.895620). Stink bugs were reared under controlled conditions (24 ±  1 °C, 60 ± 10% RH, 14:10-h photoperiod) and fed with *Phaseolus vulgaris* L. (Fabales: Fabaceae) pods. A rudimentary laboratory rearing of *N. edessae* was established from flies that emerged from adults of *E. meditabunda* collected during 2022–2024, and species identity of the flies was determined using the original descriptions (Townsend 1942) and by comparison with collection material from the Museu de Zoologia da Universidade de São Paulo (MZUSP) and type material from the Smithsonian National Museum of Natural History (NMNH). Parasitoid pupae were kept in wet vermiculite at 24 ±  1 °C, 75 ± 10% RH and a 16:8-h photoperiod until adult emergence. Adult flies were maintained in rearing cages (30 × 30 × 30 cm) under the same controlled conditions mentioned for stink bugs, with adults of *E. meditabunda* as hosts and water, sugar, raisins and commercial pollen as food.

Each of the 20 female flies was offered 27–28 adult *E. meditabunda* for 36–48 h. Each exposed host was placed in an individual container with food and kept under controlled conditions until death or parasitoid pupation. All dead hosts were dissected to detect the presence of dead parasitoid larvae inside their bodies. In all cases, the number of parasitized stink bugs (individuals with at least one larva inside their bodies, and those from which one puparium was obtained) was recorded. The number of tachinid larvae inside parasitized hosts was also quantified. The number of male and female hosts with 1, 2–4 or more than 4 larvae inside their bodies was compared in a contingency table. Additionally, photographs were taken with a binocular microscope (Leica S8APO) equipped with a camera (Leica MC120 HD) to describe the morphology of the larval instars and adult genitalia.

Parasitoid progeny (F1 = first filial generation) development times from egg to pupa and from pupa to adult were recorded. The beginning of the egg stage was defined as the moment when the parental fly was removed from the experimental unit. F1 adult flies were maintained in isolation with food and water, and their longevity was recorded. Differences in development times and longevity were evaluated considering the number of larvae inside each host (1, 2–4, more than 4), the sex of the host, and the sex of the F1 fly. For pupa to adult development time, we considered not only data from flies that emerged from the rudimentary laboratory rearing (*n* = 18), but also from field-collected parasitized stink bugs (*n* = 73) to achieve a larger number of samples. The same was done for longevity analysis, considering only the flies that survived more than 1 day (*n* = 18 from laboratory-reared hosts and *n* = 70 from field-collected hosts). Due to methodological issues, the field-collected hosts from which these parasitoids emerged could not be individualized, making it impossible to link development time data with the sex of the hosts or the number of larvae they contained. All statistical analyses were performed using the log-rank test and Kaplan-Meier curves with R Statistical Software (v4.3.2; R Core Team 2023).

## Results

Thirteen of the 20 parental flies parasitized at least one host. Almost half of the 547 stink bugs exposed to the parasitoids (268) were parasitized, and 163 pupae were obtained from those parasitized hosts (61%). Most hosts produced only a single pupa, although two pupae were obtained from the same parasitized stink bug on two occasions. Finally, 38 F1 flies completed pupal development and emerged as adults (23.31% of the obtained pupae). Of these, three flies failed to emerge successfully from their puparium; therefore, the sex of the progeny was evaluated from 13 females and 22 males. The sex ratio of the F1 (0.37 females, 0.63 males) did not differ from the 0.5 expected for each sex (*χ*^2^ = 2.31; df = 1; *p*-value = 0.13). Regarding the host, 147 females and 121 males were parasitized (Table 1), and host sex did not influence the number of parasitoid larvae per individual (*χ*^2^ = 0.29, df = 2, *p*-value = 0.86).

**Table 1:** Frequencies of *Neobrachelia edessae* larvae inside male and female *Edessa meditabunda*

Host’s sex	Larvae inside host’s body	*n*
Female	0	155
	1	56
	2–4	65
	> 4	26
Male	0	123
	1	45
	2–4	57
	> 4	19

**Fig. 1:**
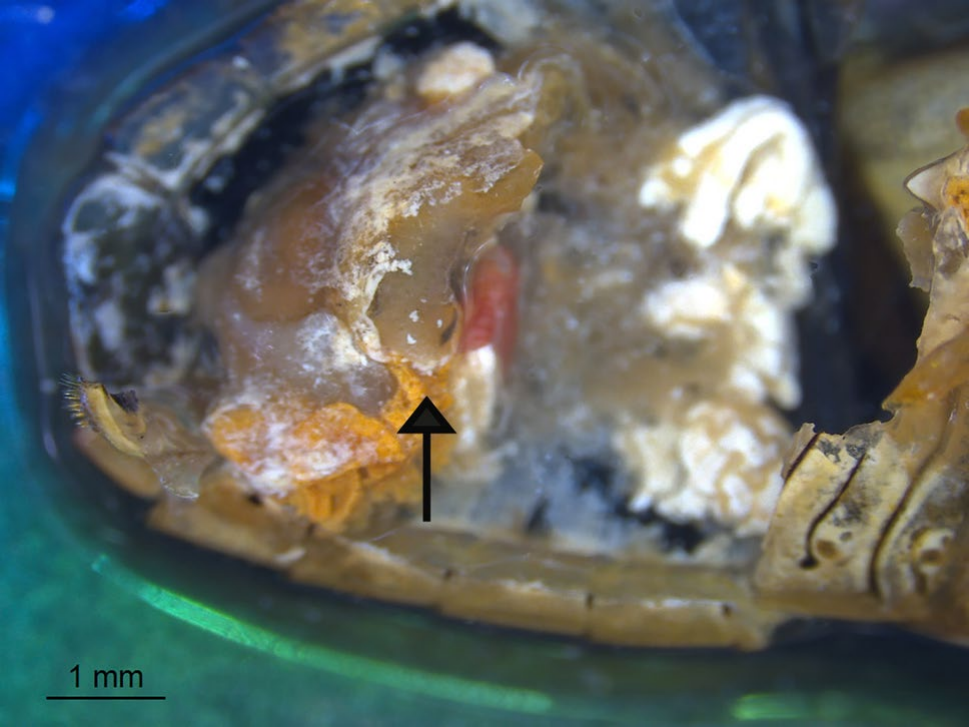
First-instar larva of *Neobrachelia edessae* inside the abdomen of *Edessa meditabunda*

**Fig. 2:**
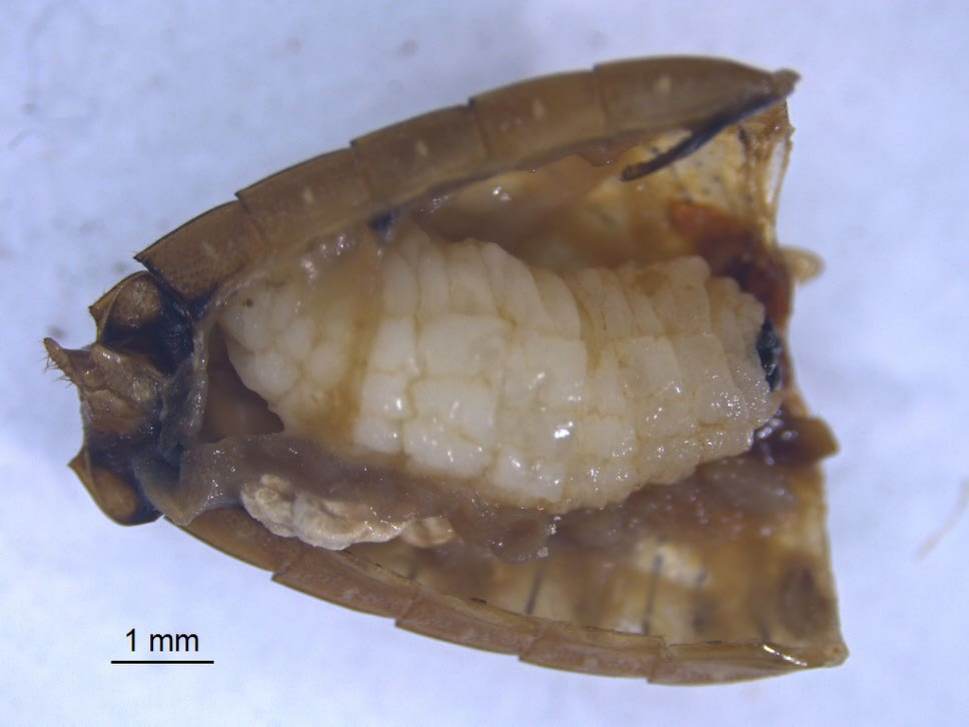
Third-instar larva of *Neobrachelia edessae* occupying most of the host abdomen

**Fig. 3:**
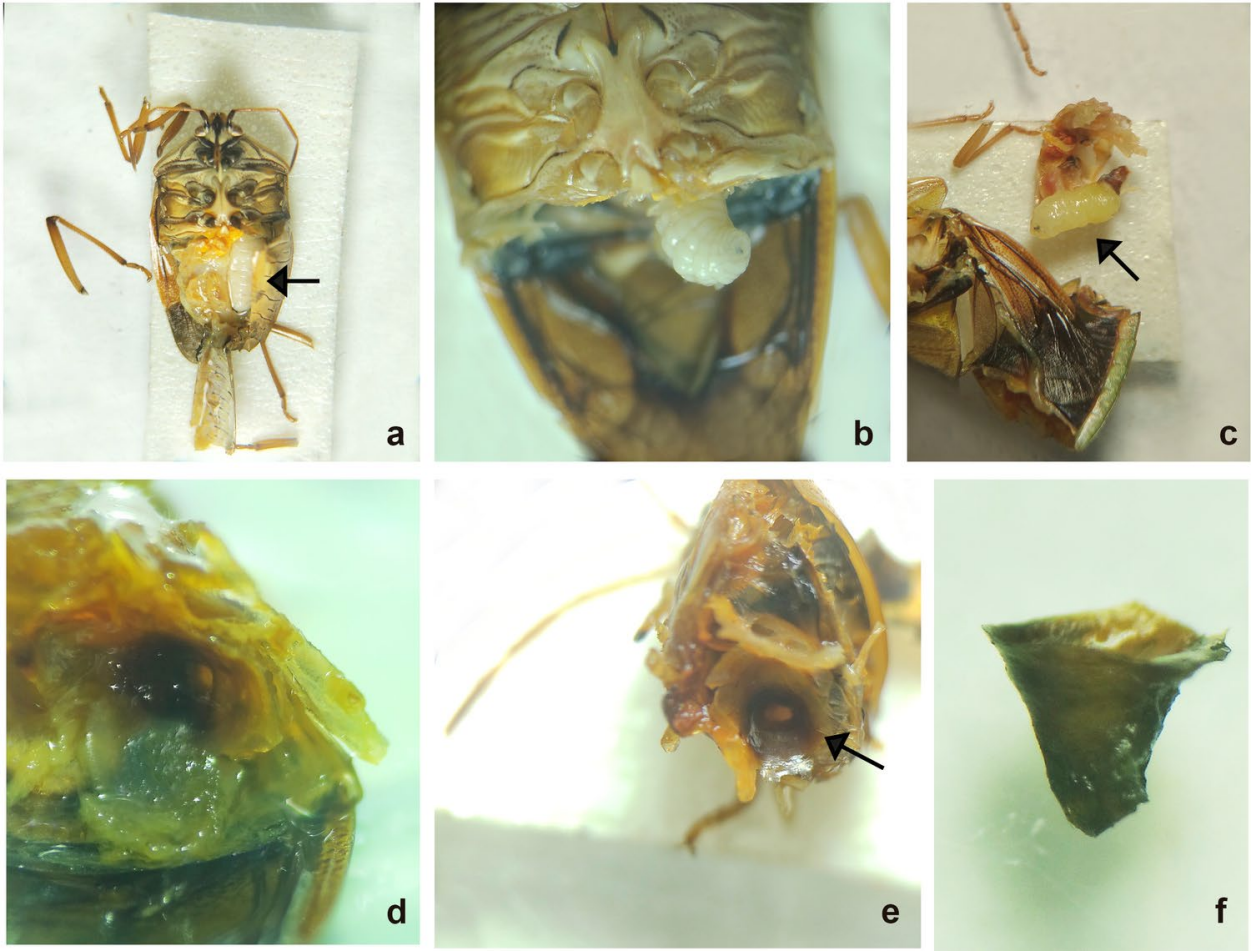
Late instars of *Neobrachelia edessae* larvae and respiratory funnels associated with their spiracles. **a** Late-second-instar larva in the host’s abdomen with spiracles pointing toward the thorax of the host. **b** Advanced larva in a host with its abdomen removed, attached via respiratory funnel to the host trachea. **c** Advanced larva removed from host, with its spiracles attached to the respiratory funnel. **d** Close-up of respiratory funnel inside host. **e** Frontal view of funnel. **f** Respiratory funnel of *Neobrachelia edessae*

We described some behaviours and morphology of the different stages of *N. edessae*. Females engaged in multiple copulation events with one or more males, each lasting approximately 20–30 min. Females often attacked the same stink bug multiple times, inserting eggs inside the body of the host with a piercing structure, as expected for Parerigonini (Dupuis 1963; Dios 2020). So, eggs were not visible externally and were not detected during dissections. First- and second-instar larvae were usually located in the abdomen of the host (Fig. 1), although some were found in the thorax near the wing muscles. All late-second- and third-instar larvae (*n* = 67) were found in the host abdomen, with their mandibles pointing toward the host genitalia and spiracles attached to a respiratory funnel connected to the host trachea (Figs. 2 and 3a, b). Respiratory funnels covered only the caudal part of the larval body and were observed only when at least one larva reached the late-second or third instar (Fig. 3c–f).

**Fig. 4:**
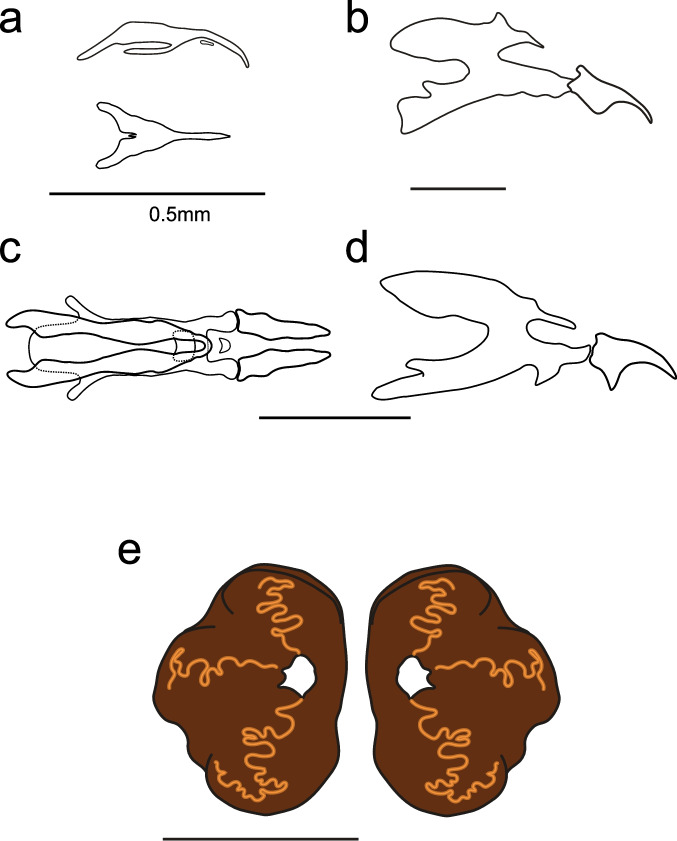
Larval structures of *Neobrachelia edessae*. **a** First-instar cephalopharyngeal skeleton, lateral and dorsal view. **b** Second-instar cephalopharyngeal skeleton, lateral view. **c** Third-instar cephalopharyngeal skeleton, dorsal view. **d** Third-instar cephalopharyngeal skeleton, lateral view. **e** Third-instar posterior spiracles. All scales 0.5 mm

The cephalopharyngeal skeleton of all three larval instars was described. Some inconspicuous short spines were observed externally in all stages, but their distribution and bands were unclear and not described. The same applies to spiracles and any accessory glands or sensilla. Only the third-instar larva’s posterior spiracle is described. The first-instar larva is cylindrical, consistent with the subfamily descriptions (Dupuis 1963), without external sclerotizations or plates, and lacking posterior appendages. The cephalopharyngeal skeleton (Fig. 4a) belongs to the first type proposed by Dupuis (1963), with a simple curved point and no articulation. The ventral cornu of the basal sclerite (“pars ventralis”) appears less developed than in other groups, and an intermediate sclerite is present.

In the second-instar larva, the cephalopharyngeal skeleton (Fig. 4b) largely agrees with Phasiinae descriptions (Dupuis 1963). It has two strong, broad, curved mouth hooks; the dorsal cornu has a posterior projection, and the ventral cornu has a dorsal projection. Hooks are articulated but closely positioned to the intermediate sclerite. The dorsal cornu projection places the larva in the first group proposed by Dupuis (1963), as also seen in Cylindromyiini and Phasiini.

The third-instar larva cephalopharyngeal skeleton (Fig. 4c, d) is similar to the second instar, but the hooks and cornua are more robust, and the articulation with the intermediate sclerite is clearer. The posterior spiracle (Fig. 4e) has a central ecdysial scar and three sinuous slits in the spiracular opening.

**Fig. 5:**
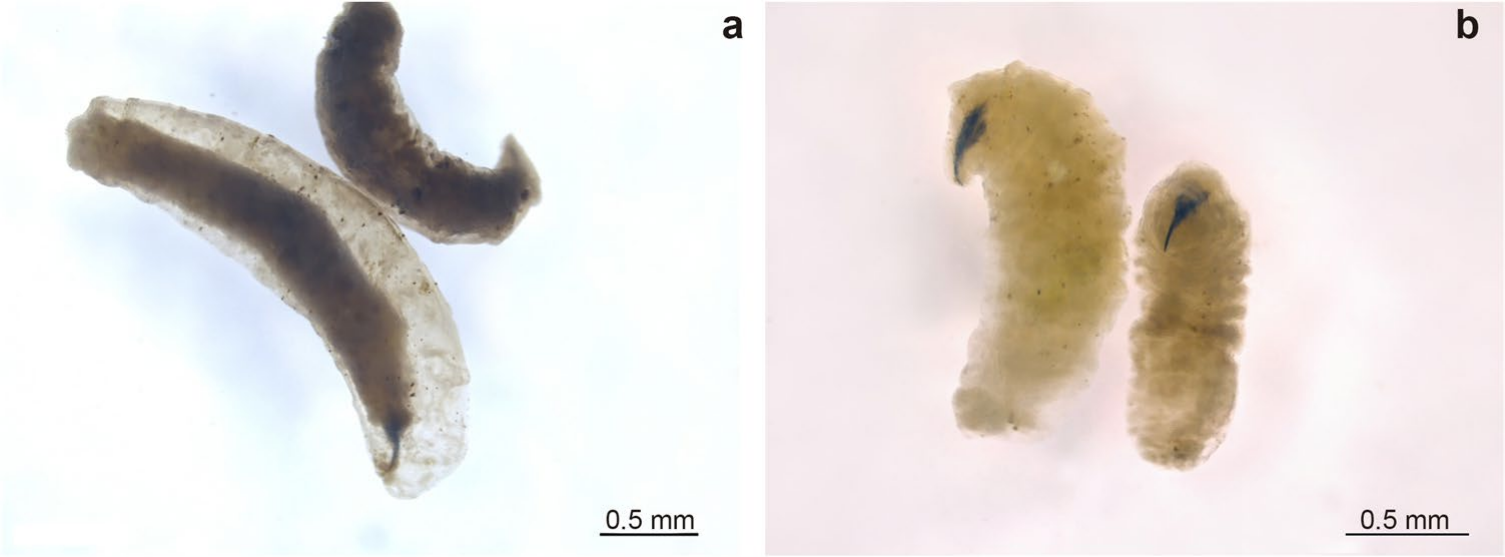
Comparison between possibly encapsulated larvae of *Neobrachelia edessae* (**a**) and healthy larvae at the same stage (**b**)

**Fig. 6:**
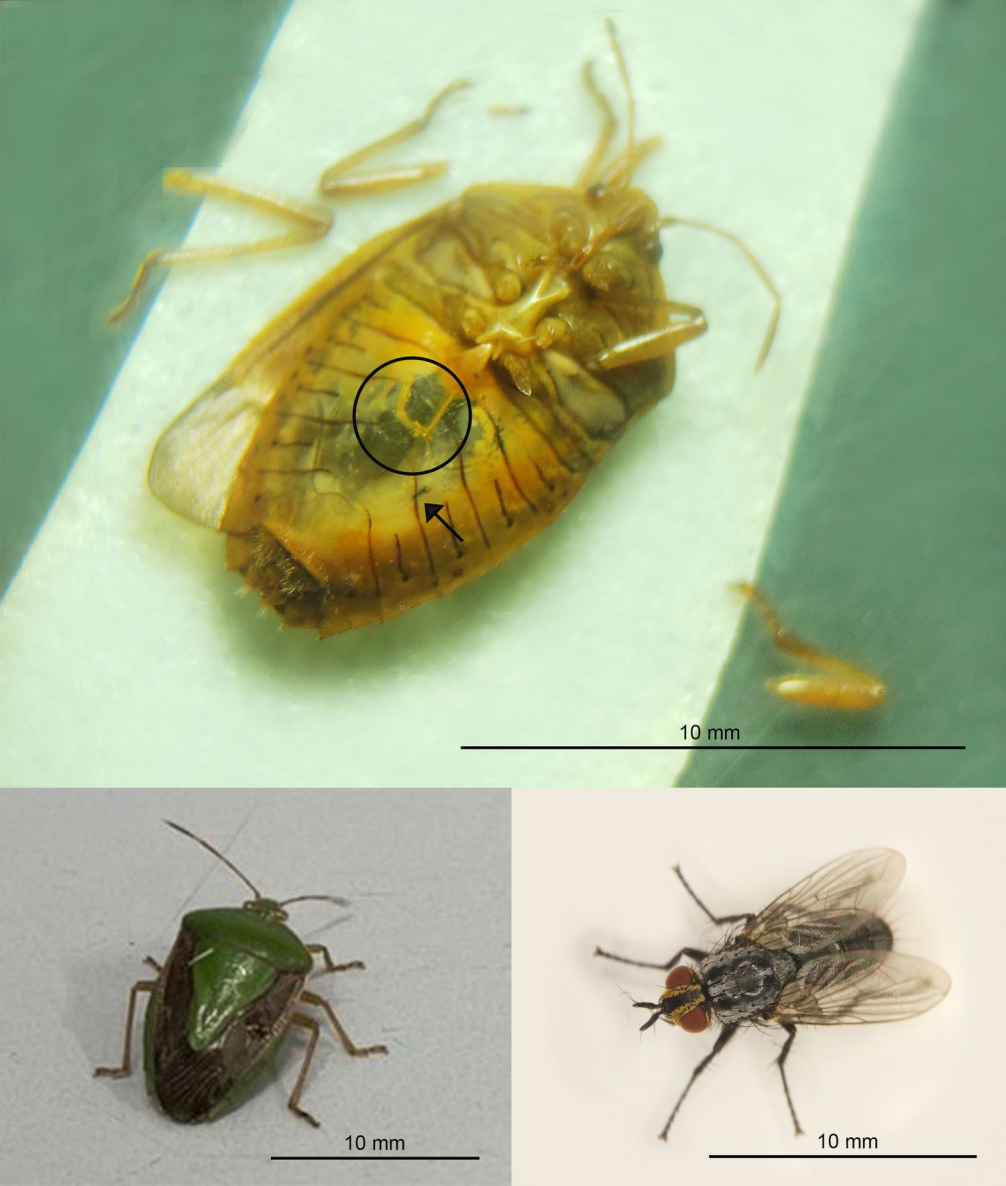
Top: ventral view of the abdomen of *Edessa meditabunda* showing translucent areas. Circle indicates removed tissue; arrow points to *Neobrachelia edessae* larval mandible. Bottom left: adult *Edessa meditabunda*. Bottom right: adult *Neobrachelia edessae*

Some early-instar larvae exhibited tissue sacs surrounding the body, pigmented foci and necrotic spots (Fig. 5a), potentially indicative of encapsulation. Small translucent zones were observed in host abdomens, likely resulting from internal scratching movements of larval mandibles (Fig. 6).

### Egg to pupa developmental time

**Fig. 7:**
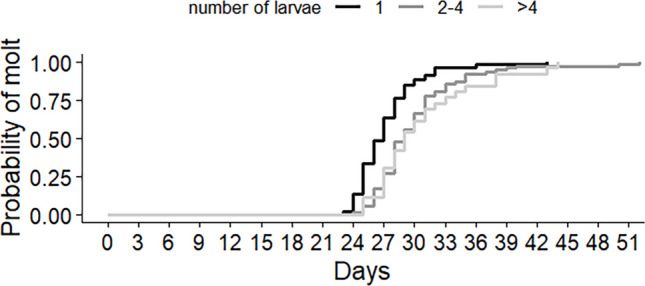
Kaplan-Meier curves representing developmental time from egg to pupa of *Neobrachelia edessae* when developing on *Edessa meditabunda* with 1, 2–4 or more than 4 parasitoid larvae per host

The number of larvae developing inside each host influenced the time required for an individual parasitoid to develop from egg to pupa (*χ*^2^ = 23.5, df = 2, *p*-value < 0.001), with the shortest development time observed when only one larva developed inside the host (27.2 days on average) (Fig. 7). Pupation was recorded from day 23 to day 32 in larvae developing solitarily. In hosts with more than one larva, pupation occurred from day 24 to day 40, on average.

No differences in egg to pupa development time were observed between flies developing inside female or male hosts (*χ*^2^ = 0.2, df = 1, *p*-value = 0.65), nor between F1 fly sexes (*χ*^2^ = 0.1, df = 1, *p*-value = 0.73) (Table 2).

**Table 2:** Developmental time from egg to pupa and from pupa to adult, and F1 longevity of *Neobrachelia edessa* developing in *Edessa meditabunda*, expressed in days (mean ± SD)

	Number of larvae inside the host			Host’s sex		F1’s sex		Host’s origin	
	1	2–4	> 4	Female	Male	Female	Male	Field	Lab
e-p	27.2 (± 0.42) (*n* = 60)	30.1 (± 0.55) (*n* = 77)	30.8 (± 1.01) (*n* = 26)	29.3 (± 0.49) (*n* = 90)	28.9 (± 0.53) (*n* = 73)	28.8 (± 0.8) (*n* = 13)	29.1 (± 0.94) (*n* = 22)	-	-
p-a	18.9 (± 0.4) (*n* = 14)	19.2 (± 0.29) (*n* = 18)	19.3 (± 0.49) (*n* = 6)	19.1 (± 0.28) (*n* = 19)	19.2 (± 0.33) (*n* = 19)	19.2 (± 0.32) (*n* = 13)	19 (± 0.28) (*n* = 22)	18.2 (± 0.34) (*n* = 73)	19.1 (± 0.2) (*n* = 56)
Longevity	11.9 (± 1.81) (*n* = 14)	9.4 (± 1.2) (*n* = 14)	13 (± 2.58) (*n* = 4)	9.2 (± 1.24) (*n* = 16)	12.6 (± 1.5) (*n* = 16)	10.1 (± 1.28) (*n* = 12)	11.4 (± 1.42) (*n* = 20)	10.2 (± 0.68) (*n* = 70)	10.1 (± 0.77) (*n* = 50)

### Pupa to adult development time

Pupal development time did not differ in relation to the number of larvae per host (*χ*^2^ = 0.3, df = 2, *p*-value = 0.85), the sex of the host (*χ*^2^ = 0.2, df = 1, *p*-value = 0.62) or the sex of the F1 flies (*χ*^2^ = 0.2, df = 1, *p*-value = 0.62). No differences were detected between flies emerged from field-parasitized versus laboratory-reared hosts (*χ*^2^ = 0.2, df = 1,* p*-value = 0.69) (Table 2).

### F1 adult flies’ longevity

Only 32 of the 38 emerged adult flies survived more than 1 day. Longevity did not differ among F1 adults that developed on hosts with one or multiple larvae (*χ*^2^ = 2.2, df = 2, *p*-value = 0.34), nor between hosts of different sexes (*χ*^2^ = 1.8, df = 1, *p*-value = 0.18). Similarly, no differences were found between sexes of F1 flies (*χ*^2^ = 0.7, df = 1, *p*-value = 0.4). Longevity also did not differ between flies from field-parasitized and laboratory-reared hosts (*χ*^2^ = 0.07, df = 1, *p*-value = 0.79) (Table 2).

## Discussion

An important number of the *N. edessae* females tested in our experiments did not accept *E. meditabunda* adults, and those that did, parasitized only about half of the available hosts. These results could indicate that the experimental conditions were not optimal for the searching and parasitism behaviours of this species of parasitoid. Self-superparasitism was frequent, with several larvae developing inside each parasitized host. According to Lack’s solution (1947), animals can control the clutch size of their progeny and evaluate environmental variables to lay an optimal number of eggs, thereby producing offspring that maximize their reproductive fitness (Adamo et al. 1995). Hymenopteran parasitoids can evaluate the quality of their hosts (i.e. size, species, etc.) and mark them after parasitising. For example, studies on *Trichogramma* sp. (Hymenoptera: Trichogrammatidae) report the ability to distinguish different host sizes and lay a certain number of eggs accordingly (van Lenteren 2003). In tachinids, however, these abilities are less developed or insufficiently studied (Adamo et al. 1995; Stireman and Shaw 2022). For instance, *Ormia ochracea* (Diptera: Tachinidae) could not adjust its clutch size according to the host size (Adamo et al. 1995). Similarly, to *N. edessae*, *O. ochracea* attacks adult hosts; however, the situation could differ for species that use caterpillars as hosts. In that last case, each different developmental stage of the maggot represents differential amounts of resources for parasitoid offspring (Caron et al. 2010). Nevertheless, very little information is available on Tachinidae in general (Cingolani et al. 2025).

In dipteran parasitoids, host selection is influenced by their oviposition strategy. We verified that *N. edessae* exhibits a direct oviposition strategy. Direct oviposition has advantages, but also limitations: tachinid flies with this strategy produce fewer eggs than species with indirect oviposition (O’Hara 2008). In this context, self-superparasitism may be considered a waste of resources, unless it confers a benefit to offspring. Self-superparasitism can increase the probability of success of the offspring (Yamada and Sugaura 2003) when the same host is attacked by another parasitoid (co-specific superparasitism). When a parasitoid female lays multiple eggs in a host, the probability of offspring success may exceed that of laying a single egg (van Alphen and Visser 1990). Additionally, laying multiple eggs may overcome the host’s immune response, as the encapsulation of the first egg may deplete hemocyte reserves, increasing survival of subsequent offspring (van Alphen and Viser 1990). Dipteran parasitoids cannot suppress host immunity or modify host physiology, as hymenopteran parasitoids do through secretions injected during oviposition (Dindo and Grenier 2023). However, tachinids have evolved other strategies to escape encapsulation, including the formation of respiratory funnels using the host immune response. Dindo and Grenier (2023) described “primary funnels” built by the first-instar larvae in the integument of the hosts, and “secondary funnels” formed by the late-first or early-second instars and associated with the tracheae or integument of the host. Besides, Komagata et al. (2024) classified respiratory funnels into “sheath-type” and “cone-type”. The first ones cover the whole body of the larva, meanwhile the second ones cover just the caudal part of the larval body. The authors suggest that the origin of both types of respiratory funnels is different and only the “sheath-type” uses the host immune response for its formation. The respiratory funnels observed in this study were always connected to the host trachea and associated with the late-second or third larval instar, corresponding to the secondary type. It only covered the terminal part of the larval body, like the “cone-type”, as observed among other Phasiinae, such as some Gymnosomatini (Dupuis 1963; Komagata et al. 2024). Moreover, we evidenced some larvae trapped in flocculent masses, similar to the “sheath-type” respiratory funnels, but always dead in first- or early-second instar, likely corresponding to encapsulation events. The larvae of the third stage that we found dead during dissections always looked healthy, with a “shiny” aspect and without symptoms of necrosis or cell aggregation around their bodies. Parasitoids trapped in an encapsulation event usually look opaque (Brodeur and Vet 1995). However, we note that microscopic observations may limit the detection of encapsulation, which is usually assessed histologically (Valigurová et al. 2014; Luna et al. 2016).

The lack of host-marking ability likely contributes to frequent self-superparasitism in tachinids. Visual cues may be important for host location (Stireman et al. 2006), but the specific cues used by *N. edessae* remain unknown. Tachinids with direct oviposition may also respond to plant-host chemical cues (Dindo and Grenier 2023), although studies are mostly on parasitoids of caterpillars. Evaluating the first trophic level’s influence on *N. edessae* is interesting.

Intrinsic competition is relevant given the high frequency of self-superparasitism. In our study, larvae developing alone reached pupation sooner than those developing with conspecifics, likely due to sibling competition. Resource limitation increases with larval density, delaying pupation when multiple larvae share a host. In contrast to solitary larvae, hosts with several larvae may not allow any individual to reach the nutritional threshold for moulting as quickly.

While some tachinid species show reduced development times with increased larval density (King et al. 1976; Ziser et al. 1977; Caron et al. 2010), our results align with Reitz (1995), who observed longer development in superparasitized larvae. To the best of our knowledge, there is not much information about development times of Phasiinae species dealing with superparasitism. This study is an important first approximation to this subject. Additionally, evidence of signs of necrosis and dark points around many larvae were registered, possibly corresponding to melanised wounds that may have originated in a physical battle between competing siblings, as described in Reitz (1995). Tachinids display several mechanisms to eliminate supernumerary larvae inside a host, from physical combat to anoxia (Reitz 1995), but the exact mechanisms in *N. edessae* remain unknown.

Neither host sex nor F1 fly sex affected egg to pupa development in *N. edessae*. Some phasiines use male pheromones of their heteropteran hosts to find them (Zarbin et al. 2012), and this could be reflected in a preference for male hosts. Higaki and Adachi (2011) exposed male and female flies of *Gymnosoma rotundatum* (Diptera: Tachinidae) to adult females of *Plautia stali* (Hemiptera: Pentatomidae), including females baited and not baited with a synthetic pheromone. They registered parasitism only in females baited with the pheromone. However, they found that in natural conditions, both sexes of the host were parasitized (Higaki and Adachi 2011). It could be expected that male and female hosts offer different resources for parasitoid larvae due to their anatomical differences (i.e. reproductive systems) and to the size of their bodies, as in some stink bug species, males are smaller than females (Moura and Gonzaga 2019). However, in our study, *N. edessae* showed no preference between male and female hosts, and development times of the parasitoid were not influenced by the sex of the exploited host. It is interesting to note that under controlled conditions, unparasitized stink bugs survive on average 100 days, while parasitized individuals survive for about 50 days (Barakat 2025).

Besides, differences in development times between male and female flies of several tachinid species could be related to the size of each sex of the adults of the parasitoids themselves, and the resources they need to complete their development (Kalyebi and Nakamura 2006). For example, Caron et al. (2010) found that females of *Compsilura concinnata* (Diptera: Tachinidae) were usually heavier than males and needed more resources to complete their development. Here, we found no difference in development times between parasitoid sexes. It is not clear whether the population’s sex ratio in the wild shows the same trend.

According to van Lenteren (2003), parasitoids emerge as adults with a limited supply of energy. If the amount of food ingested by the larva is scarce, either due to the effects of competition or to poor host quality, energy reserves for the developing larva will be low, and development times could be affected. The quality of the host could also affect the size, longevity and fecundity of parasitoid offspring (van Lenteren 2003). If the host is not good enough, the energy reserves could be low, and this could potentially modify the development times in the life cycle and the longevity of the offspring. Since pupal development and adult longevity did not differ between lab-reared and field-parasitized hosts, our lab-reared *E. meditabunda* appear suitable for *N. edessae* development. The same was observed with the longevity of adults. None of the variables evaluated in this study significantly affected the lifespan of the adult parasitoid offspring. However, the number of emerged adults was low relative to the number of pupae obtained, highlighting that the pupal stage is a critical point of the life cycle, and external factors like humidity affect adult emergence. More understanding of this topic is necessary to establish a mass rearing of *N. edessae*. Here, we present a first approximation of laboratory rearing of this species.

Considering that only two species have been recorded as hosts of *N. edessae* to date (Guimarães 1977), it would be interesting to explore whether this parasitoid can establish a host-parasitoid interaction with other stink bug species. Many species within the tachinid group continue to expand their host range over time (Markova 1999; Mückstein et al. 2007; Gudin et al. 2024). Morphological characterization is essential to avoid misidentification and improve understanding of parasitoid biology, ecology and biocontrol potential. Until now, there were no studies addressing the natural history and development of the tribe Parerigonini. This tribe is basally positioned among Phasiinae (Blaschke et al. 2018; Dios 2020), and studying its biology contributes to understanding evolutionary patterns.

In the Neotropics, the use of tachinids as biocontrol agents has great potential because of their high biodiversity, but the development of biological control programs faces several challenges (van Lenteren et al. 2025). Information on the use of tachinids as biological control agents in Latin America is scarce and fragmented (Cingolani et al. 2025). Tachinids have been intensively used to control sugarcane stem borers (Aya et al. 2019) and stink bugs globally, but basic biology remains poorly known. Overcoming these barriers is essential for advancing biocontrol programs. Consequently, augmentative biocontrol is presently suboptimal, whereas conservation strategies are currently more feasible (Fernández et al. 2024). In this sense, the preservation of natural enemies like tachinid flies in the field is a priority, but more knowledge on the topic is necessary. This work constitutes a first approximation to the interaction between *N. edessae* and *E. meditabunda*. In the future, more efforts are needed to exploit the great potential that this parasitoid species could have.

## Data Availability

Data are available from the corresponding author upon reasonable request.

## References

[CR1] Adamo SA, Robert D, Perez J, Hoy RR (1995) The response of an insect parasitoid, *Ormia ochracea* (Tachinidae), to the uncertainty of larval success during infestation. Behav Ecol Sociobiol 36(2):111–118. 10.1007/BF00170716

[CR2] Araújo VA, Bacca T, Dias LG (2020) Anatomy of male and female reproductive organs of stink bugs pests (Pentatomidae: Heteroptera) from soybean and rice crops. Biota Neotrop 20(4):e20201045. 10.1590/1676-0611-bn-2020-1045

[CR3] Aya VM, Montoya-Lerma J, Echeverri-Rubiano C, Michaud JP, Vargas G (2019) Host resistance to two parasitoids (Diptera: Tachinidae) helps explain a regional outbreak of novel *Diatraea* spp. stem borers (Lepidoptera: Crambidae) in Colombia sugarcane. Biol Control 129:18–23. 10.1016/j.biocontrol.2018.11.009

[CR4] Barakat MC (2025) Redescubriendo interacciones poco exploradas: potencialidades y limitantes de los parasitoides del estado adulto de chinches fitófagas (Hemiptera: Pentatomidae) como agentes de control biológico. Dissertation, Universidad Nacional de La Plata, La Plata, Argentina. 10.35537/10915/181812. Accessed 22 Apr 2026

[CR5] Barakat MC, Díaz SP, Dios R, Cingolani MF (2023) Biology of the parasitoid fly *Neobrachelia edessae* (Diptera: Tachinidae) on *Edessa meditabunda* (Hemiptera: Pentatomidae). 7IEIC ebook of oral sessions abstracts. https://bicyt.conicet.gov.ar/fichas/produccion/11727425. Accessed 22 Apr 2026

[CR6] Beckers OM (2022) Parasitism of the katydid *Neoconocephalus triops* (Orthoptera: Tettigoniidae) by the tachinid flies *Ormia lineifrons* and *Neomintho* sp. (Diptera: Tachinidae). Fla Entomol 105(2):133–136

[CR7] Blaschke JD, Stireman JO III, O’Hara JE, Cerretti P, Moulton JK (2018) Molecular phylogenetics and piercer evolution in the bug-killing flies (Diptera: Tachinidae: Phasiinae). Syst Entomol 43:218–238. 10.1111/syen.12272

[CR8] Brodeur J, Vet LEM (1995) Relationships between parasitoid host range and host defence: a comparative study of egg encapsulation in two related parasitoid species. Physiol Entomol 20(1):7–12. 10.1111/j.1365-3032.1995.tb00794.x

[CR9] Caron V, Myers JH, Gillespie DR (2010) The failure to discriminate: superparasitism of *Trichoplusia ni* Hübner by a generalist tachinid parasitoid. Bull Entomol Res 100(3):255–261. 10.1017/S0007485309990198 19586578 10.1017/S0007485309990198

[CR10] Cingolani MF, Barakat MC, Cerretti P, Chirinos DT, Ferrer F, Gaviria Vega J, Grenier S, Kondo T, Pape T, Plowes R, Salas J, Vargas G, Whitmore D, Dindo ML (2025) Dipteran parasitoids as biocontrol agents. Biocontrol 70(3):285–300. 10.1007/s10526-025-10317-1

[CR11] Conti E, Avila G, Barratt B, Cingolani F, Colazza S, Guarino S, Hoelmer K, Laumann RA, Maistrello L, Martel G, Peri E, Rodriguez-Saona C, Rondoni G, Rostás M, Roversi PF, Sforza RFH, Tavella L, Wajnberg E (2021) Biological control of invasive stink bugs: review of global state and future prospects. Entomol Exp Appl 169:28–51. 10.1111/eea.12967

[CR12] de Aquino MFS, Sujii E, Moraes MCB, Borges M, Laumann RA (2020) Diversidade e incidência de parasitoides de percevejos adultos na cultura da soja e sua relação com o uso de inseticidas. EMBRAPA-Recursos Genéticos e Biotecnologia, Boletim De Pesquisa e Desenvolvimento 364:24

[CR13] de Salvo CP, Salazar L, González M, Schling M, Muñoz G, Rondinone G, Le Pommellec M (2025) Desarrollo sostenible de la agricultura en América Latina y el Caribe: Desafíos y oportunidades. Inter-American Development Bank. 10.18235/0013382

[CR14] Dellapé PM, Melo MC, Dellapé G, Olivera L (2025) Pentatomomorpha (Hemiptera: Heteroptera) species from Argentina and Uruguay. Available at https://biodar.unlp.edu.ar/pentatomomorpha/ [Accessed March 26, 2026]

[CR15] Dindo ML, Grenier S (2023) Production of dipteran parasitoids. In: Morales-Ramos JA, Rojas MG, Shapiro-Ilan DI (Eds) Mass production of beneficial organisms, 2nd edn. Academic Press, pp 71–100 10.1016/B978-0-12-822106-8.00003-8

[CR16] Dindo ML, Rezaei M, De Clercq P (2019) Improvements in the rearing of the tachinid parasitoid Exorista larvarum (Diptera: Tachinidae): influence of adult food on female longevity and reproduction capacity. J Insect Sci 19(2):1–6. 10.1093/jisesa/iey122 10.1093/jisesa/iey122 PMC6403477 30822779

[CR17] Dios RVP (2020) Cladistic analysis of Phasiinae (Diptera: Tachinidae), based on morphological characters [Doutorado em Zoologia, Universidade de São Paulo]. 10.11606/T.41.2020.tde-11052020-154956

[CR19] Duncan MW (2017) Determinants of host use in tachinid parasitoids (Diptera: Tachinidae) of stink bugs (Hemiptera: Pentatomidae) in Southwest Ohio. Dissertation, Wright State University, Dayton, OH. https://etd.ohiolink.edu/acprod/odb_etd/etd/r/1501/10?clear=10&p10_accession_num=wright1495723449203563. Accessed 22 Apr 2026

[CR20] Dupuis D (1963) Essai monographique sur les Phasiinae (Diptères Tachinaires parasites d’Héteroptères). Mém Mus Natl Hist Natl Sér A Zool 26:1–454

[CR21] Fernández CA, Punschke EL, Cingolani MF, Carrizo AP, Barakat MC, De Vilhena Perez Dios R, Blengino F, Huarte F, Montero GA (2024) Tachinids in conservation biological control of phytophagous Pentatomidae. Biocontrol 69(5):539–550. 10.1007/s10526-024-10282-1

[CR22] Godfray HCJ, Partridge L, Harvey PH (1991) Clutch size. Annu. Rev. Ecol. Syst, 22, 409–429. http://www.jstor.org/stable/2097268. Accessed 13 June 2025

[CR23] Gudin FM, Campos LDD, Redü DR, De Mello FDAG (2024) Parasitoid flies (Diptera, tachinidae) in true crickets (Orthoptera, grylloidea): new host records from Brazil, identification key to parasitoids, and revision of host-parasitoid interactions. J Orthopt Res 33(1):41–58. 10.3897/jor.33.108456

[CR24] Guimarães JH (1977) Host-parasite and parasite-host catalogue of South American Tachinidae (Diptera). Arq Zool 28(3):1–131. 10.11606/issn.2176-7793.v28i3p1-131

[CR25] Harvey JA, Poelman EH, Tanaka T (2013) Intrinsic inter- and intraspecific competition in parasitoid wasps. Annu Rev Entomol 58(1):333–351. 10.1146/annurev-ento-120811-153622 23092242 10.1146/annurev-ento-120811-153622

[CR26] Higaki M, Adachi I (2011) Response of a parasitoid fly, Gymnosoma rotundatum (Linnaeus) (Diptera: Tachinidae), to the aggregation pheromone of Plautia stali Scott (Hemiptera: Pentatomidae) and its parasitism of hosts under field conditions. Biol Control 58:215–21. 10.1016/j.biocontrol.2011.05.009

[CR27] Kalyebi A, Nakamura S (2006) The biology of the parasitoid fly *Drino inconspicuoides* (Diptera: Tachinidae) in the host *Mythimna separata* (Lepidoptera: noctuidae). Appl Entomol Zool 41(2):365–370. 10.1303/aez.2006.365

[CR28] King EG, Miles LR, Martin DF (1976) Some effects of superparasitism by Lixophaga diatraeae of sugarcane borer larvae in the laboratory. Entomol Exp Appl 20(3):261–269. 10.1111/j.1570-7458.1976.tb02642.x

[CR29] Komagata S, Ogawa K, Tachi T (2024) The bug-killer fly *Gymnosoma rotundatum* (L.) (Diptera: Tachinidae) forms the respiratory funnel independently of the host’s immune response. Bull Entomol Res 114(3):424–432. 10.1017/S0007485324000221 38629304 10.1017/S0007485324000221

[CR30] Lack D (1947) The significance of clutch-size. Ibis 89:302–352

[CR31] Liljesthröm GG, Ávalos DS (2015) Nuevas asociaciones entre Phasiinae (Diptera: Tachinidae) y Pentatomidae (Hemiptera: Heteroptera) fitófagos en la pampa ondulada (Argentina) y descripción del macho de *Dallasimyia bosqi* Blanchard. Rev Soc Entomol Arg 74(3–4):145–152. http://www.scielo.org.ar/scielo.php?script=sci_arttext&pid=S0373-56802015000200006&lng=es&tlng=es. Accessed 13 Jun 2025

[CR32] Lucini T, Panizzi AR, Dios RVP (2020) Tachinid fly parasitism and phenology of the Neotropical Red-Shouldered Stink Bug, Thyanta perditor (F.) (Heteroptera: Pentatomidae), on the Wild Host Plant, Bidens pilosa L. (Asteraceae). Neotrop Entomol 49:98–107. 10.1007/s13744-019-00706-4 31347023 10.1007/s13744-019-00706-4

[CR33] Luna MG, Desneux N, Schneider MI (2016) Encapsulation and self-superparasitism of Pseudapanteles dignus (Muesebeck) (Hymenoptera: Braconidae), a parasitoid of Tuta absoluta (Meyrick) (Lepidoptera: gelechiidae). PLoS One 11(10):e0163196. 10.1371/journal.pone.0163196 27732609 10.1371/journal.pone.0163196 PMC5061380

[CR34] Markova TO (1999) New host and distribution data of tachinid flies of subfamily Phasiinae (Diptera, tachinidae) in Siberia and russian far east. Far east entomol https://www.biosoil.ru/FEE/Publication/93. Accessed 13 Jun 2025

[CR35] Mohamad R, Goubault M (2023) Self-superparasitism, oviposition order, and delay: does offspring survival explain host exploitation strategies of females under interspecific competition in a solitary parasitoid wasp? Entomol Exp Appl 171(10):765–771. 10.1111/eea.13343

[CR36] Mohammadi ZM, Clay PM, Feeney R, Harmath P, Keshavarz M, Gunderson MA (2020) Characterization of farmers’ management practices and strategies: a comparison between Argentine and U.S. farmers. Int Food Agribus Manag Rev 23(2):235–252. 10.22434/IFAMR2019.0158

[CR37] Moura RR, Gonzaga MO (2019) Spatial variation in sex ratio and density explains subtle changes in the strength of size-assortative mating in *Edessa contermina* (Hemiptera: Pentatomidae). Acta Oecol 95:86–92. 10.1016/j.actao.2018.12.003

[CR38] Mückstein P, Tschorsnig HP, Vaňhara J, Michalková V (2007) New host and country records for European Tachinidae (Diptera). Entomol Fenn 18(3):179–183. 10.33338/ef.84396

[CR39] O’Hara JE (2008) Tachinid flies (Diptera: Tachinidae). pp. 3675–3686. In: Capinera JL (ed) Encyclopedia of Entomology, 2nd edn. Springer, Netherlands, Dordrecht, p 4346

[CR40] O’Hara JE, Henderson SJ, Wood DM (2020) Preliminary checklist of the Tachinidae of the world. Version 2.1. PDF document, 1039 pages. http://www.nadsdiptera.org/Tach/WorldTachs/Checklist/Worldchecklist.html. Accessed 13 Jun 2025

[CR41] Pajač Beus M, Lemić D, Skendžić S, Čirjak D, Pajač Živković I (2024) The brown marmorated stink bug (Hemiptera: Pentatomidae). A major challenge for global plant production. Agric Eng 14:13–22. 10.3390/agriculture14081322

[CR42] Panizzi AR (2015) Growing problems with stink bugs (Hemiptera: Heteroptera: Pentatomidae): species invasive to the U.S. and potential neotropical invaders. Am Entomol 61:223–233. 10.1093/ae/tmv068

[CR43] Panizzi AR, Lucini T, Aldrich JR (2022) Dynamics in pest status of phytophagous stink bugs in the Neotropics. Neotrop Entomol 51:18–31. 10.1007/s13744-021-00928-5 35028921 10.1007/s13744-021-00928-5

[CR44] Parker HL (1953) Miscellaneous notes on South American dipterous parasites. Boll Lab Entomol Agrar F Silvestri. Portici 12:45–73

[CR45] R Core Team (2023) R: A Language and Environment for Statistical Computing. R Foundation for Statistical Computing, Vienna, Austria

[CR46] Reitz S (1995) Superparasitism and Intraspecific Competition by the Solitary Larval-Pupal Parasitoid Archytas marmoratus (Diptera: Tachinidae). Fla Entomol 78(4):578–585. 10.2307/3496043

[CR47] Reitz SR, Trumble JT (1997) Effects of linear furanocoumarins on the herbivore *Spodoptera exigua* and the parasitoid *Archytas marmoratus*: host quality and parasitoid success. Entomol Exp Appl 84(1):9–16. 10.1046/j.1570-7458.1997.00192.x

[CR48] Roitberg B, Bernhard P (2008) State‐dependent problems for parasitoids: case studies and solutions.In: Wajnberg E, Bernstein C, van Alphen J (eds) Behavioral ecology of insect parasitoids: from theoretical approaches to field applications. Wiley-Blackwell, Oxford, pp 335–356

[CR49] Silva RAD, Degrande PE, Pereira MDC, Souza EPD (2021) Temporal variation and spatial distribution of the pest insect *Edessa meditabunda* in cotton (*Gossypium hirsutum*) as an alternative host plant. Rev Bras Entomol 65:e20210029

[CR50] Stenberg JA, Sundh I, Becher PG et al (2021) When is it biological control? A framework of definitions, mechanisms, and classifications. J Pest Sci 94:665–676. 10.1007/s10340-021-01354-7

[CR51] Stireman, JO, Shaw SR (2022) Natural history and ecology of caterpillar parasitoids. In: Marquis RJ, Koptur S (eds) Caterpillars in the middle. Springer International Publishing, pp 225–272 10.1007/978-3-030-86688-4_8

[CR52] Stireman JO, O’Hara JE, Wood DM (2006) Tachinidae: evolution, behavior, and ecology. Annu Rev Entomol 51:525–55. 10.1146/annurev.ento.51.110104.151133 16332222 10.1146/annurev.ento.51.110104.151133

[CR53] Stireman JO, Cerretti P, O’Hara JE, Blaschke JD, Moulton JK (2019) Molecular phylogeny and evolution of world Tachinidae (Diptera). Mol Phylogenet Evol 139:106358. 10.1016/j.ympev.2018.12.002 30584917 10.1016/j.ympev.2018.12.002

[CR54] Stireman JO, Cerretti P, O’Hara JE, Moulton JK (2021) Extraordinary diversification of the “bristle flies” (Diptera: Tachinidae) and its underlying causes. Biol J Linn 133(1):216–236. 10.1093/biolinnean/blab010

[CR55] Townsend CHT (1942) Two new reared South American flies. Rev Entomol 13:438–439

[CR56] Valigurová A, Michalková V, Koník P, Dindo ML, Gelnar M, Vaňhara J (2014) Penetration and encapsulation of the larval endoparasitoid Exorista larvarum (Diptera: Tachinidae) in the factitious host Galleria mellonella (Lepidoptera: pyralidae). Bull Entomol Res 104(2):203–212. 10.1017/S0007485313000655 24355436 10.1017/S0007485313000655

[CR57] van Lenteren J (ed) (2003) Quality control and production of biological control agents: Theory and testing procedures, 1st ed. CABI Publishing. 10.1079/9780851996882.0000

[CR58] van Alphen JJ, Visser ME (1990) Superparasitism as an adaptive strategy for insect parasitoids. Annu Rev Entomol 35:59–79. 10.1146/annurev.en.35.010190.000423 2405774 10.1146/annurev.en.35.010190.000423

[CR59] van Lenteren J, Bueno VHP, Luna MG, Colmenárez Y (2020) Biological control in latin america and the Caribbean: information sources, organizations, types and approaches in biological control. Biological Control in Latin America and the Caribbean: Its Rich History and Bright Future 1–20. 10.1079/9781789242430.0001

[CR60] van Lenteren J, Bueno VHP, Bettiol W (2025) Latin America has the largest area under augmentative biological control worldwide, mainly with applications in open field crops. Biol Control 207:105827. 10.1016/j.biocontrol.2025.105827

[CR61] Yamada YY, Sugaura K (2003) Evidence for adaptive self-superparasitism inthe dryinid parasitoid *Haplogonatopus atratus* when conspecifics are present. Oikos 103:175–181

[CR62] Zarbin PHG, Fávaro CF, Vidal DM, Rodrigues MACM (2012) Male-produced sex pheromone of the stink bug *Edessa meditabunda*. J Chem Ecol 38(7):825–835. 10.1007/s10886-012-0144-4 22692411 10.1007/s10886-012-0144-4

[CR63] Ziser SW, Wojtowicz JA, Nettles WC (1977) The effects of the number of maggots per host on length of development, puparial weight, and adult emergence of Eucelatoria sp. Ann Entomol Soc Am 70(5):733–736. 10.1093/aesa/70.5.733

